# Editorial: Surgical outcomes in acute care surgery: should we introduce the concept of time-critical condition?

**DOI:** 10.3389/fsurg.2023.1234200

**Published:** 2023-07-26

**Authors:** Pietro Fransvea, Antonio La Greca, Francesco Giovinazzo, Gianluca Costa, Gabriele Sganga

**Affiliations:** ^1^ Emergency Surgery and Trauma, Fondazione Policlinico Universitario A. Gemelli, IRCCS Roma - Università Cattolica del Sacro Cuore, Rome, Italy; ^2^ Unit of General and Liver Transplant Surgery, Fondazione Policlinico Universitario A. Gemelli, IRCCS, Università Cattolica del Sacro Cuore, Rome, Italy; ^3^ Colorectal Surgery Unit, Surgery Center, Fondazione Policlinico Universitario Campus Bio-Medico, University Campus Bio-Medico of Rome, Rome, Italy

**Keywords:** outcomes, surgery, acute care, precision surgery, morbidity

**Editorial on the Research Topic** Acute care surgery, emergency surgery, surgical outcomes

## Introduction

Patients undergoing Emergency Gastrointestinal Surgery (EGS) are so heterogeneous in terms of procedures they receive, comorbidities and physiologic derangement at the time of diagnosis and treatment that perioperative risk stratification, surgical planning and risk mitigation may be exceedingly cumbersome. Nonetheless, EGS, while representing about 15% of the whole surgical burden worldwide, still accounts for more than 50% of global surgical morbidity and mortality (2). Thus, a structured decision-making process, with accurate risk stratification and patients' priority addressing (3–5), seems even more crucial in the emergency than in the elective setting. On one hand, we need to better assess EGS patients' needs and improve surgical outcomes in the emergency setting. How? First, perioperative risk stratification is crucial to improve shared decision-making among the care team and the patient, perioperative planning, and risk mitigation (5, 8). As suggested by Yun Il, several factors are thought to be related to the postoperative outcome, but frailty has recently gained increased attention and its preoperative screening has been advocated as a critical tool in predicting length of stay, operative risk, and surgical outcomes in the elderly (1). Second, as reported by Eydivandi N, a thorough attention in intraoperative care items, such as levels of carbon dioxide insufflation, analgesia, i.v. fluid load, has a pivotal role in preventing post-operative surgical complications. In this respect, the so called “Enhanced recovery after emergency surgery” concept may represent the new frontier of perioperative care for EGS patients (6, 7). Finally, as reported by Ali et al., the Clavien–Dindo classification still plays a crucial role in assessing postoperative complications and predicting the impact of surgery on quality of life (8). On the other hand, the new emerging concept of EGS as a time dependent condition can help achieving early diagnosis and treatment, thus improving outcomes and reducing health care costs (9, 10). During the COVID19 pandemic, we have witnessed a dramatic decrease in surgical emergencies but also an increase in the severity of acute diseases, as patients, frightened by the risk of contagion, went late to the ED. Definitive treatment was often postponed in favour of a non-operative treatment (NOM), both due to concomitant Sars-Cov-2 infection in these patients as well as to shortage of resources (such as ICU beds and surgical rooms) engaged with COVID-19 patients (11). If we look at a single disease such as acute appendicitis, several studies have shown that a conservative approach may further increase the risk of recurrence: more than one third of the patients treated by antibiotics only have been re-admitted for a recurrent episode of appendicitis (12–16). Moreover, a systematic review and metanalysis by Podda et al. showed that appendectomy remains the most effective treatment for patients with uncomplicated acute appendicitis and that antibiotics-first strategy for uncomplicated acute appendicitis in adults is associated with increased rates of peritonitis at surgery (16). Coming to the general landscape of EGS, it is well known that a prolonged waiting time from the onset of symptoms to surgical treatment is directly related to an increased risk of major bowel resection and postoperative complications (17–19). Peritonitis due to gastric perforation is associated with an increasing risk of mortality of 2% for each hour of delay in surgical treatment after the diagnosis (20–23). Patients with acute cholecystitis should undergo surgery within a time lapse of less than 10 days, such as stated in modern guidelines (24–29). Femur fractures should receive a definitive surgical treatment within 48 h from diagnosis, as this time cut-off is directly linked to better outcomes (30). Delay in the treatment of acute diseases is not only of ethical interest in terms of harm for the patient, but also has a critical impact in terms of social costs arising from the disability and reduced productivity of the injured. This concept is worldwide accepted for the so-called time-dependent acute diseases such as stroke, acute myocardial infarction and polytrauma: maybe we should think about acute abdominal pain as a time-dependent disease also. Based on the evidence of the literature and common surgeons' experience, we hypothesized a “Traffic Light System” (TLS) for patient waiting in the ED with surgical diseases needing an operative treatment based on the waiting time since arrival. When a diagnosis is done, the patient enters in the “green light” status and is ready for surgery. If surgery cannot be performed timely, patients enter in a “yellow light” status, acquiring priority over other diseases. “Red light” means that the patient cannot wait anymore and needs a prompt treatment. With this simple stratification system, waiting times for surgery in the emergency department may decrease together with the need for major resections, morbidity and mortality rates. Last but not least, new technologies such as robotic platforms and machine learning models, though affected by high costs, can help achieve better outcomes in EGS. In this context, a paradigm shift may be represented by the application of the so-called “precision surgery” concept to acute care surgery: a global effort in education and practice to ensure an elevated standard of care to all acute care surgery patients in terms of knowledge, technical skills, technology and commitment leading to a reduction in healthcare costs, including costs for chronic post-surgical disability (31, 32).
